# Demographics and risk for containment surgery in patients with unilateral Legg–Calvé–Perthes disease: a national population-based cohort study of 309 patients from the Swedish Pediatric Orthopedic Quality Register

**DOI:** 10.2340/17453674.2024.40907

**Published:** 2024-06-18

**Authors:** Miriam G WADSTRÖM, Nils P HAILER, Yasmin D HAILER

**Affiliations:** Section of Orthopaedics, Department of Surgical Sciences, Uppsala University, Sweden

## Abstract

**Background and purpose:**

It is controversial as to which patients affected by Legg–Calvé–Perthes disease (LCPD) benefit from containment surgery. This population-based study based on data from a national quality registry aims to assess the incidence of LCPD and to explore which factors affect the decision for surgical intervention.

**Methods:**

This observational study involved 309 patients with unilateral LCPD reported between 2015 and 2023 to the Swedish Pediatric Orthopedic Quality Register (SPOQ). Descriptive statistics and logistic regression models were used for analysis.

**Results:**

In 2019, the assessed incidence of LCPD in the Swedish population of 2–12-year-olds was 4.2 per 10^5^. 238 (77%) were boys with a mean age of 6 years. At diagnosis, 55 (30%) were overweight or obese, rising to 17 patients (39%) and 16 patients (40%) at 2-year follow-up for surgically and non-surgically treated groups, respectively. At diagnosis, affected hips had reduced abduction compared with healthy hips, and their abduction remained restricted at the 2-year follow-up. Surgically treated patients had inferior abduction compared with non-surgically treated ones at diagnosis. The adjusted risk for containment surgery increased with age and in the presence of a positive Trendelenburg sign but decreased with greater hip abduction.

**Conclusion:**

We found a lower national yearly incidence (4.2 per 10^5^) than previously reported in Swedish studies. A higher proportion of overweight or obese patients compared with the general Swedish population of 4–9-year-olds was identified. Increasing age, positive Trendelenburg sign, and limited hip abduction at diagnosis correlated with increased surgical intervention likelihood.

Legg–Calvé–Perthes disease (LCPD) is a relatively rare childhood hip disorder that greatly affects hip joint health in adult life [1]. Given the variable presentation of LCPD and its potential long-term consequences, identifying factors that influence the decision for surgery is crucial. Exploring clinical and radiographic patterns could aid in identifying patients who would benefit from surgical intervention [2]. The incidence of LCPD varies greatly globally, ranging from 0.4 to 24.4 per 100,000 individuals annually [3]. In Sweden, the reported incidence is between 8.5 and 9.3 per 100,000 children annually [4,5]. Due to its scarcity, conducting prospective large-scale studies on this disorder is difficult, but in the absence of better evidence, population-based research can fill some knowledge gaps. Such information may prove useful in the clinical evaluation of LCPD patients, including assessment of prognosis and choice of treatment strategies.

This study aims to explore: (i) the incidence, and (ii) patient characteristics and factors influencing the choice to perform containment surgery for patients with unilateral LCPD, based on data provided by the Swedish Pediatric Orthopedic Quality Register (SPOQ).

## Methods

### Study design and data source

This population-based national longitudinal cohort study analyzed data gathered from SPOQ. This registry was initiated in 2015, to establish a national framework for long-term monitoring of patients with pediatric orthopedic diseases and thus provides a unique possibility to fill knowledge gaps in the realm of LCPD. With the information provided by SPOQ, potential associations between clinical features, radiographic development, and treatment choices can be identified. This study was conducted according to the STROBE guidelines for observational studies [6].

### Study population

The study population included all patients diagnosed with LCPD between the ages of 2 and < 13 years at diagnosis, starting in 2015 and ending on January 24, 2024.

### Swedish Pediatric Orthopedic Quality Register

SPOQ includes the diagnoses of Legg–Calvé-Perthes disease, slipped capital femoral epiphysis, developmental dysplasia of the hip, pes equinovarus adductus, and patellar dislocation. The sub-registry for patients with Legg–Calvé–Perthes disease follows a structured approach for long-term monitoring. SPOQ has guidelines for internal validation of the registry through logical data controls, regular sub-register reviews, national and targeted coverage analyses, regional data checks, quality improvement meetings, expert radiographic reviews, and central data assessments to ensure high data accuracy and reliability.

### Variables

The study cohort consisted of patients diagnosed with LCPD included in SPOQ. The presence or absence of surgical treatment was our main outcome. We also investigated the progress of the disease over time. Potential associations of baseline variables, physiological parameters (age, sex, body mass index [BMI]), clinical parameters (range of motion in the hip joint), the severity of the disease (lateral pillar classification), at the time of diagnosis (if possible to classify) and after a 2-year follow-up, with the choice of surgical treatment. Additionally, Reimer’s migration percentage (i.e., the horizontal fraction [%] of the femoral head that is uncovered by the acetabulum) was registered at the 2-year follow-up to assess hip displacement.

### Statistics

For descriptive statistics, means, medians, ranges, and frequencies were used. To assess the incidence rate of LCPD, we divided all new cases of LCPD reported in SPOQ by the total resident population of 2–12-year-olds in Sweden in 2019 retrieved from Statistics Sweden. According to the Swedish National Board of Health and Welfare, SPOQ had a completeness of 70% for LCPD patients in 2019. The incidence rate was presented as the number of new LCPD patients per 100,000 in 2019. The chi-square test was used to evaluate distributions of categorical variables and Student’s t-test was used to analyze differences between means.

The risk of undergoing surgical treatment was estimated by odds ratios (OR) with 95% confidence intervals (CI) based on logistic regression models including age, sex, lateral pillar class, hip abduction, and BMI. Surgical treatment was defined as containment surgery but excluded diagnostic procedures or apophysiodesis of the greater trochanter. Only patients with unilateral involvement were included in the analysis, as several factors present in patients with bilateral disease might confound our results, such as the potentially different etiology of bilateral vs unilateral LCPD. In addition, the exclusion of patients with bilateral involvement avoided dependency issues. Statistical analyses were performed using R statistical software, version 4.3.2, and R studio, version 2023.12.1+402 (R Foundation for Statistical Computing, Vienna, Austria).

### Ethics, data sharing, funding, and disclosures

This study was conducted following the guidelines of the Helsinki Declaration and after approval by the Swedish Ethical Review Authority (May 31, 2022; approval number 2022-02369-01 and January 30, 2024; approval number 2023-07307-01). The study was financed by grants from the Swedish state under the agreement between the Swedish government and the county councils, the ALF agreement. The authors declare no competing interests. Complete disclosure of interest forms according to ICMJE are available on the article page, doi: 10.2340/17453674.2024.40907

## Results

### Outline of the study population

334 patients and 359 hips were registered at the time of diagnosis. 309 patients had unilateral involvement. Of these, 253 (82%) had been in the registry for a minimum of 2 years and 201 hips (79%) had a registered follow-up visit 2 years after the diagnosis (Figure).

**Figure:**
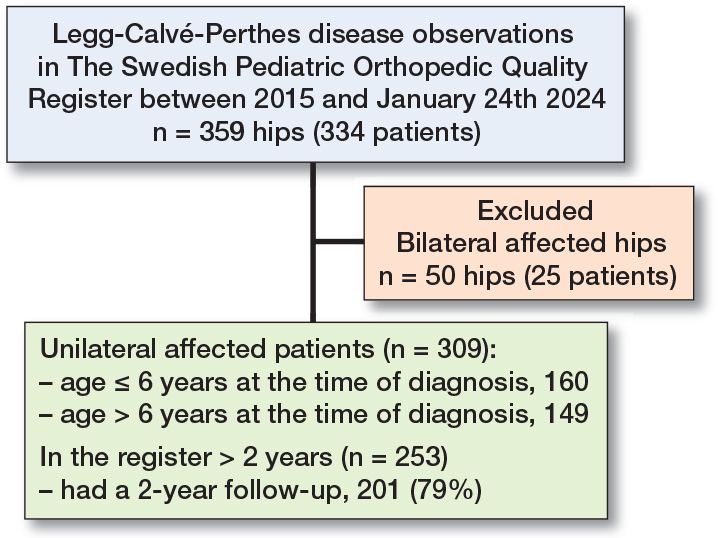
Flowchart illustrating the study population.

The long-term monitoring of the patients included in SPOQ comprises the collection of baseline demographic information together with clinical and radiographic assessments performed at the time of diagnosis, 2 years post-diagnosis, and at the ages of 10, 13, and 18 years. Additional information on whether and what type of surgical treatment was performed is collected.

### Incidence of LCPD in Sweden

Based on the SPOQ register we found an incidence of 3.2 LCPD cases per 10^5^ individuals. Because the SPOQ had a completeness of only 70% in 2019, the assessed incidence of LCPD cases in the entire Swedish population of 2–12-year-olds in 2019 was 4.2 cases per 10^5^ individuals.

### Clinical parameters at the time of diagnosis and at 2-year follow-up in patients with unilateral LCPD

Of the 309 patients with unilateral LCPD reported to the SPOQ, 238 patients (77%) were boys, the mean age at diagnosis was 6 (2–13) years, and 86 patients (29%) presented with a positive Trendelenburg sign at the time of diagnosis (Table 1).

**Table 1:** The study population at the time of diagnosis consisting of patients with unilateral LCPD. The “surgically treated” column details the characteristics of patients who later underwent surgical treatment. Values are count (%) unless otherwise specified

Factor	All n = 309	Surgically treated n = 120	Non-surgically treated n = 189
Sex			
Female	71 (23)	34 (28)	37 (20)
Male	238 (77)	86 (72)	152 (80)
Age, mean (range)	6 (2–13)	7 (3–13)	5 (2–13)
Age group			
< 5	108 (35)	20 (17)	88 (47)
5–8	141 (46)	65 (54)	76 (40)
> 8	60 (19)	35 (30)	25 (13)
Trendelenburg positive	86 (29)	49 (42)	37 (20)
Missing data	10	5	5

Most patients, 132 (71%), had a BMI within the range of under- or normal weight at the time of diagnosis and 55 patients (29%) were overweight or obese. At the 2-year follow-up, 17 patients (39%) in the surgically treated group and 16 patients (40%) in the non-surgically treated group were overweight or obese. At the time of diagnosis, the affected hips showed a statistically significant reduction in abduction compared with healthy hips, with an average lag of 15°. At the 2-year follow-up, the affected hips still exhibited a statistically significant inferior abduction, with an average lag of 10° compared with healthy hips. Patients who underwent surgery had more limited abduction at the time of diagnosis than those who did not undergo surgery. However, by the 2-year follow-up, the difference in abduction range between the healthy and affected hips had improved for both surgically treated and non-surgically treated patients (Table 2).

**Table 2:** Clinical parameters of patients with unilateral LCPD (309 hips) at the time of diagnosis and at 2-year follow-up. The “surgically treated” column details the group of patients who underwent surgical treatment. Values are count (%) unless otherwise specified

Factor	All at diagnosis n = 309	Surgically treated		Non-surgically treated	
		at diagnosis n = 120	at 2-year follow-up n = 84	at diagnosis n = 189	at 2-year follow-up n = 117
BMI					
Under- to normal weight	132 (71)	50 (68)	27 (61)	82 (72)	25 (61)
Overweight	35 (19)	12 (16)	11 (25)	23 (20)	8 (20)
Obese	20 (11)	11 (15)	6 (14)	9 (8)	8 (20)
Missing data	122	47	40	75	76
Abduction (°) mean (range)	39 (0 to 90)	33 (0 to 80)	34 (–20 ^a^ to 70)	42 (10 to 90)	47 (8 to 80)
Abduction difference (°)^b^	15	20	15	13	6

a –20° indicates 1 patient with adduction contracture.

b Average difference in abduction, healthy vs. affected side.

### Radiographic parameters at the time of diagnosis and at 2-year follow-up in patients with unilateral LCPD

At the time of diagnosis, 147 (48%) of the hips were too early in the course of their disease to be classified according to the lateral pillar classification. We expect this group to represent Elizabethtown class 1a–2a. At the 2-year follow-up, all patients could be classified, and in the surgically treated group, 26 (37%) were classified as lateral pillar B, 15 (21%) were classified as B/C, and 26 (37%) as C. In the non-surgically treated group, 46 (39%) were classified as lateral pillar B, 17 (15%) were classified as B/C, and 24 (21%) as C at the 2-year follow-up. Reimer’s migration percentage was within normal range at the 2-year follow-up, in both the surgically and non-surgically treated group (Table 3).

**Table 3:** Radiographic parameters at the time of diagnosis and the 2-year follow-up in patients with unilateral affected hips. Values are count (%) unless otherwise specified

Factor	All	Surgically treated		Non-surgically treated	
	at diagnosis n = 309	at diagnosis n = 120	at 2-year follow-up n = 84	at diagnosis n = 189	at 2-year follow-up n = 117
Lateral pillar					
A	38 (12)	12 (10)	4 (6)	26 (14)	14 (12)
B	77 (25)	32 (27)	26 (37)	45 (24)	46 (39)
B/C	26 (8)	16 (13)	15 (21)	10 (5)	17 (15)
C	19 (6)	8 (7)	26 (37)	11 (6)	24 (21)
Before fragmentation					
stage ^a^	147 (48)	52 (43)	NA	95 (51)	NA
Missing data	2 (1)	–	13 (15)	2 (1)	16 (14)
Reimer’s migration percentage ^b^	–	–	20	–	20

a Elizabethtown classification 1a–2a.

b Reimer’s migration percentage, normal range: 17–27%.

### Estimated risk for containment surgery in patients with unilateral LCPD

The adjusted risk for containment surgery increased with age (OR 1.3, CI 1.1–1.6) and with the presence of a positive Trendelenburg sign at the time of diagnosis (OR 4.1, CI 1.8–9.9), whereas it decreased with increasing hip abduction (OR 0.95, CI 0.92–0.98). In univariable analysis, female sex, overweight or obesity, and patients with lateral pillar B/C showed a trend for an increase in the risk of containment surgery, but not statistically significant in the crude or adjusted analysis (Table 4).

**Table 4:** Patient factors at the time of diagnosis associated with risk for containment surgical treatment, analyzed by logistic regression models in 309 patients with unilateral affected hips

Patient characteristics	OR (CI)	Adjusted OR ^a^ (CI)
Age ^b^	1.1 (0.9–1.3)	1.3 (1.1–1.6)
Sex		
Male	Ref.	Ref.
Female	1.3 (0.7–2.3)	1.0 (0.4–2.4)
Lateral pillar		
B	Ref.	Ref.
B/C	1.4 (0.5–3.3)	0.6 (0.1–2.6)
C	0.6 (0.2–1.7)	0.9 (0.1–4.7)
Before fragmentation stage	0.7 (0.4–1.3)	0.9 (0.4–2.1)
Hip abduction ^b^	0.97 (0.95–0.99)	0.95 (0.92–0.98)
Trendelenburg sign positive	2.7 (1.6–4.7)	4.1 (1.8–9.9)
BMI		
Under- to normal weight	Ref	Ref
Overweight	1.1 (0.4–2.6)	0.9 (0.3–2.4)
Obese	1.4 (0.4–3.8)	1.3 (0.3–5.0)

a Adjusted for age, sex, hip abduction, lateral pillar class, and BMI.

b Continuous variable, OR per one-unit change.

## Discussion

### Incidence and demographics

Our study assessed the incidence of LCPD in Sweden in 2019 to be 4.2 cases per 10^5^ individuals. The reported yearly incidence ranges from 0.4 to 24.4 per 10^5^ across different populations, influenced by factors such as geographic location, ethnicity, and socioeconomic status. We found the incidence in Sweden to be lower than previously reported in Sweden. For instance, the Moberg study in 1992 found an annual incidence of 8.5 cases per 10^5^ children aged 0–14 in the Uppsala region [4]. Similarly, the study by Johansson et al. reported an incidence of LCPD of 9.3 per 10^5^ children born between 1973 and 1993, using data from the Swedish Patient Register [5]. The lower incidence in our study can be explained by the strict inclusion criteria of the SPOQ, where only patients with a Swedish personal identification number between the ages of 2 and 12 years are included, to exclude atypical cases and to enable follow-up. In contrast, the Swedish Patient Register risks including patients with secondary femoral head necrosis, e.g., after a fracture, chemotherapy, or coagulation disorders, misdiagnosed as LCPD.

### Patient characteristics

As expected, the majority of patients diagnosed with LCPD were boys, consistent with previous studies [3]. The typical onset of LCPD occurs around the age of 6 years, falling within the well-established age range of 4 to 8 years old [3]. While there has not been a significant shift in the typical age range for LCPD diagnosis, recent advancements in diagnostic techniques, such as magnetic resonance imaging (MRI) and advanced radiographic methods, have improved our ability to detect subtle early changes in the femoral head [2]. This increased sensitivity, coupled with greater awareness among healthcare providers and parents, may contribute to earlier diagnosis in some cases. However, it is important to note that the mainstay of LCPD diagnosis remains clinical evaluation, including assessment of symptoms and physical examination findings, complemented by plain radiography.

Even though LCPD is predominantly a disease among boys, girls seem more commonly to present with a more severe form of LCPD [7]. Some authors suggest that hormonal factors might be the cause [7,8]. Yet, in our material, we could not see this pattern. The severity of LCPD and age at onset were similar between the 2 sexes in our cohort, and we could not see a statistically significant difference in risk for surgical treatment in girls compared with boys (Table 5, see Appendix).

**Table 5:** Sub-group analysis of female and male patients: clinical and radiographic parameters at the time of diagnosis and the 2-year follow-up. Values are count (%) unless otherwise specified

Factor	Female		Male	
	at diagnosis n = 71	at 2-year follow-up n = 49	at diagnosis n = 238	at 2-year follow-up n = 152
BMI				
Under- to normal weight	26 (62)	13 (57)	106 (73)	39 (62)
Overweight	9 (21)	7 (30)	26 (18)	12 (19)
Obese	7 (17)	3 (13)	13 (9)	11 (18)
Missing data	29 (41)	26 (53)	93 (39)	90 (59)
Abduction (°) mean (range)	37 (0 to 80)	40 (20 to 70)	39 (0 to 90)	42 (–20 ^a^ to 80)
Abduction difference^b^	18	9	15	10
Lateral pillar				
A	6 (8)	4 (10)	32 (14)	14 (11)
B	22 (31)	18 (45)	55 (23)	54 (41)
B/C	8 (11)	8 (20)	18 (8)	24 (18)
C	3 (4)	10 (25)	16 (7)	40 (30)
Before fragmentation stage ^c^	32 (45)	NA	115 (49)	
Missing data	–	9 (18)	2 (1)	20 (13)
Reimer’s migration percentage ^d^	–	22	–	20

a –20° indicates 1 patient with abduction contracture.

b Average difference in abduction, healthy vs affected side.

c Elizabethtown classification 1a–2a.

d Reimer’s migration percentage, normal range: 17–27%.

### Childhood obesity

Historically, LCPD patients were described as “undersized at the time of developing Perthes’ disease and remained shorter than average throughout life” [9]. In recent studies, however, an increasing incidence of obesity among LCPD patients is described [10-13]. In agreement with these studies, between 30% and 40% of the children in our cohort were overweight or obese, a notably higher percentage compared with the general population of children in Sweden. The Public Health Agency of Sweden estimates that 13% of 4-year-olds and 23% of 6–9-year-olds are overweight or obese in Sweden today [14,15]. It can be argued that the increased weight in these children could be due to activity restrictions related to the symptoms and therapy; however, childhood overweight and obesity are multifactorial rather than only being explained by inactivity due to the diagnosis [16,17]. Further, patients in our study already were overweight or obese at the time of diagnosis. The proportion of overweight or obese patients did not change significantly over time, suggesting that these conditions were likely pre-existing and not a consequence of activity restrictions [18]. Obesity is a well-known risk factor for several musculoskeletal conditions, including LCPD [10,11]. The increased weight load on the hip joint in obese children increases the stress on the hip joint and may exacerbate the symptoms and delay the healing process [19].

### Clinical presentation and diagnosis

At the time of diagnosis, we found that 147 (48%) of patients were too early in the course of their disease to be classified according to the lateral pillar classification. This proportion of patients with an unclassifiable stage is explained by the fact that it usually takes about 7 months until the fragmentation stage, in which the lateral pillar classification is applicable [20,21]. It is therefore of interest to introduce a method to identify patients who will already develop a more severe form of LCPD (lateral Pillar B, B/C, or C) during the early stages.

Clinically, patients with LCPD often present with hip pain, limping, stiffness, and a limited range of motion. In our study, 86 patients (29%) had a positive Trendelenburg sign at diagnosis, indicating an increased risk for surgical treatment. The Trendelenburg sign assesses hip muscle strength and function, providing crucial information regarding the impact of LCPD on the hip abductor muscles. This non-invasive test is valuable for ongoing evaluation. Additionally, reduced hip joint abduction is essential for assessing LCPD severity, disease progression, and treatment outcomes. Both a positive Trendelenburg sign and reduced abduction are linked to poorer patient-reported outcomes and serve as prognostic indicators, aligning with existing literature [19,22]. Our findings support this, showing an elevated surgical risk in cases of decreased abduction and a positive Trendelenburg sign.

### Treatment

We found that 120 (39%) overall, and 38 (32%) hips in children under the age of 6, were treated surgically. These results present a deviation from international practices, where surgical interventions among children aged below 6 years are infrequent. The reason for this deviation is unclear. In Sweden, non-weight-bearing treatment regimens are typically not applied [23]. The lower threshold for surgical treatment compared with global standards even in younger children could reflect a consequence of this. The prognosis and choice of treatment for LCPD are mainly determined by the severity of the disease and the age of the patient. Other than age, no major differences in patients characteristics of the 2 age groups were present (Table 6, see Appendix). Generally, patients with an onset of the disease under 6 years of age have a favorable prognosis [24] while older children with limited remodeling potential often have poorer outcomes [25]. Patients at which age benefit from surgery is still under discussion internationally [24,25]. Regardless of age, surgery should be initiated before the advanced stages of the disease. The objective is to restore the sphericity of the femoral head and ensure its congruency within the acetabulum to mitigate further damage, promote remodeling, and prevent complications associated with the disease [28-30].

**Table 6:** Sub-group analysis of patients 6 years or younger and older than 6 years: clinical and radiographic parameters at the time of diagnosis and the 2-year follow-up. Values are count (%) unless otherwise specified

Factor	≤ 6 years old		> 6 years old	
	at diagnosis n = 160	at 2-year follow-up n = 101	at diagnosis n = 149	at 2-year follow-up n = 100
BMI				
Under- to normal weight	63 (68)	25 (57)	69 (73)	27 (66)
Overweight	18 (19)	7 (16)	17 (18)	12 (29)
Obese	12 (13)	12 (27)	8 (9)	2 (5)
Missing data	67 (42)	57 (56)	55 (37)	59 (59)
Abduction (°) mean (range)	45 (10 to 90)	47 (20 to 80)	32 (0 to 80)	35 (–20 ^a^ to 70)
Abduction difference (°) ^b^	11	6	20	14
Lateral pillar				
A	19 (12)	13 (13)	19 (13)	5 (7)
B	37 (23)	48 (48)	40 (27)	24 (33)
B/C	9 (6)	12 (12)	17 (11)	20 (27)
C	14 (9)	26 (26)	5 (3)	24 (33)
Before fragmentation stage ^c^	79 (50)	NA	68 (46)	NA
Missing data	2 (1)	2 (2)	–	27 (27)
Reimer’s migration percentage ^d^	–	20	–	21

a–d See Table 5.

### Factors associated with containment surgery

Surgical treatment in patients with LCPD has been suggested to be more successful than nonoperative treatment in patients older than 8 years old and with lateral pillar B or B/C [31]. In our study, we identified increasing age and a positive Trendelenburg sign at the time of diagnosis as associated factors for containment surgical treatment. Previous studies suggest that those with more severe disease (lateral pillar B/C or worse) benefit from surgical treatment [26]. However, we did not detect any statistically significant association with the lateral pillar class and containment surgery. The final treatment outcome can first be assessed at skeletal maturity. Given that our follow-up was limited to 2 years, we cannot determine these outcomes from our data.

We found that a greater hip abduction was associated with a decreased risk of surgical treatment. This observation may be indicative of a common characteristic in more severe forms of LCPD, which often manifests with restricted hip joint mobility. The inverse relationship between the risk of surgery and hip abduction underscores the potential clinical relevance of assessing hip joint mobility as a prognostic indicator for treatment decisions in cases of LCPD. Our findings align with those of Perry et al., who noted that decisions regarding surgery were primarily based on hip stiffness and the child’s age. However, the significance of joint stiffness as a key prognostic factor could not be demonstrated [32].

### Limitations

First, registries such as SPOQ rely on input from healthcare professionals, which can lead to errors and missing data. Non-mandatory measures may also affect the validity of the findings. Second, measurement of hip abduction is examiner-dependent, which could possibly affect the quality of input data. However, SPOQ provides detailed instructions, and previous studies show excellent agreement in hip abduction measurements [33]. Third, factors influencing the choice of surgical treatment, such as individual treatment preferences, surgeon experience, and socioeconomic factors, are not fully captured in the registry. This study is descriptive and based on a classification system that does not consider intervention timing, limiting establishing direct causal relationships. Fourth, despite a 100% national coverage of SPOQ [34], the registry has 70% completeness compared with the Swedish patient register. However, the Swedish patient register may overestimate LCPD prevalence due to misclassification. Finally, loss of follow-up is a limitation, primarily due to a small number of patients with 2-year follow-up.

### Conclusion

We found a lower national yearly incidence (4.2/10^5^) than previously reported in Swedish studies. The patients in this study presented a trend towards a younger age at diagnosis of LCPD than previously reported in Sweden, alongside an increased prevalence of overweight and obese individuals compared with the general population. Factors such as increased age, a positive Trendelenburg sign, and limited hip joint abduction at the time of diagnosis were identified as indicators associated with a greater probability of surgical intervention.

In perspective, these findings can serve as a foundation for conducting future prospective studies aimed at optimizing treatment strategies and improving outcomes for LCPD patients.

## Notes

MGW: Designed the study, undertook statistical analysis, analyzed results, and drafted the manuscript. NPH: Reviewed and edited the manuscript for clarity, style, and content. YDH: Designed the study, undertook statistical analysis, analyzed results, and reviewed and edited the manuscript.

The authors would like to express their gratitude to Ewa Freyhult for statistical proofreading and consulting. They also thank the registered units for their meticulous data collection in the Swedish Pediatric Orthopedic Quality (SPOQ) register and the participating patients for their contribution to this study.

Handling co-editors: Ilkka Helenius and Robin Christensen

*Acta* thanks Jeffrey Kessler and other anonymous reviewers for help with peer review of this manuscript.
